# Fish and Fish Oil Intake in Relation to Risk of Asthma: A Systematic Review and Meta-Analysis

**DOI:** 10.1371/journal.pone.0080048

**Published:** 2013-11-12

**Authors:** Huan Yang, Pengcheng Xun, Ka He

**Affiliations:** 1 Institute of Toxicology, Third Military Medical University, Chongqing, China; 2 Department of Epidemiology, Gillings School of Global Public Health, University of North Carolina at Chapel Hill, Chapel Hill, North Carolina, United States of America; 3 Department of Nutrition, Gillings School of Global Public Health and School of Medicine, University of North Carolina at Chapel Hill, North Carolina, United States of America; 4 Department of Epidemiology and Biostatistics, School of Public Health, Indiana University, Bloomington, Indiana, United States of America; The Ohio State Unversity, United States of America

## Abstract

Although laboratory studies suggest that long-chain n-3 polyunsaturated fatty acids (LCn3PUFAs) may reduce risk of asthma, epidemiological data remain controversial and inconclusive. We quantitatively reviewed the epidemiological studies published through December 2012 in PubMed and EMBASE by using a fixed-effects or random-effects model. Eleven studies, comprised of 99,093 individuals (3,226 cases), were included in the final dataset. Of them, 7 studies examined associations between intake of fish or LCn3PUFA and risk of asthma: 4 studies in children (996 cases from 12,481 children) and 3 in adults (1,311 cases from 82,553 individuals). Two studies (69 cases from 276 infants) investigated LCn3PUFA levels in mothers’ milk, and two studies assessed maternal fish consumption (786 cases from 2,832 individuals) during lactation and/or plasma LCn3PUFA levels during pregnancy (64 cases from 951 infants) in relation to offspring’s asthma. The pooled relative risk of child asthma were 0.76 (95% CI, 0.61–0.94) for fish consumption and 0.71 (95% CI, 0.52–0.96) for LCn3PUFA intake. No statistically significant association was found in studies among adults. Epidemiological data to date indicate that fish or LCn3PUFA intake may be beneficial to prevent asthma in children. Further studies are needed to establish causal inference and to elucidate the potential mechanisms.

## Introduction

Asthma, a chronic inflammation of the airways that results in narrowing of the bronchial tubes [1], has increased dramatically over the past three decades all around the world, in both adults and children [2]–[4]. According to the data from US center of disease control (CDC), the prevalence of asthma increased from 7.3% to 8.4% in the US over the first 10 years of this century [5], and it affected 235 million people worldwide in 2011 with an increasing prevalence [6]. This situation leads to a considerable economic burden in both direct and indirect medical costs. It has been suggested that there may be an additional 100 million people who may suffer from asthma by 2025 [6]. Therefore, identifying potential protective or risk factors of asthma is of great public health significance.

Since the laboratory studies suggest that asthma is an inflammatory process, it has been hypothesized that high intake of long-chain n-3 polyunsaturated fatty acids (LCn3PUFAs) may be beneficial to preventing asthma. In the past decades, a number of epidemiological studies have examined the association between the intake of fish or LCn3PUFAs and the risk of asthma [7]–[15]. However, the findings from these studies were inconsistent. Two cohort studies that recruited 3,595 and 3,086 participants, respectively, found that people who ate fish more than once per week had their risk of asthma lowered significantly by 6% to 45% as compared with non-consumers [15], [16]. Another cohort study reported a 16% risk reduction in fish consumers compared with non-consumers, though it was statistically non-significant [17]. One case-control study found a non-significant risk reduction comparing the highest fish consumption group with the lowest [18], while one cross-sectional study found fish consumption was positively associated with the risk of asthma when comparing participants who consumed fish 1–2 servings/week with those who consumed 1–2 servings/month [19]. A few randomized clinical trials (RCTs) have been published. One trial reported beneficial effect of fish oil supplementation on asthma [27].

To provide an integrated review and a reliable quantitative assessment of the association between the intake of fish and LCn3PUFAs and the risk of asthma, we conducted a systematic review and meta-analysis of prospective cohort studies as well as RCTs with the existing data.

## Methods

### Study Selection

The meta-analysis was performed based on the checklist of the Meta-analysis of Observational Studies in Epidemiology [20]. All prospective cohort studies published in English-language journals from 1966 to December 2012, which reported the association between fish or fish oil intake/biomarker and incidence of asthma, were identified by searching PubMed using MESH words “(((((("Fish Oils" [Mesh]) OR "Fishes" [Mesh]) OR "Fatty Acids, Omega-3" [Mesh]) OR "Seafood" [Mesh]) AND "Asthma" [Mesh])) OR (((((n-3 fatty acids) OR fish) OR fish oil) OR sea foods) AND asthma)” or using free words ((((n-3 fatty acids) OR fish) OR fish oil) OR sea foods) AND asthma” and by searching EMBASE using Emtree words “omega 3 fatty acid'/exp OR 'fish'/exp OR 'fish oil'/exp OR 'sea food'/exp AND asthma'/exp” or using free words “'n-3 fatty acids' OR 'fish' OR fish oils' OR 'sea foods' AND 'asthma.'” Additional information was retrieved through Google and a hand search of the references from relevant articles.

Two of our authors (HY and PX) independently reviewed all relevant papers and identified eligible studies. Discrepancies were resolved by group discussion. A study would be included if it was a prospective cohort design, and the relative risks (RRs) and corresponding 95% confidence intervals (CIs) of asthma relating to fish and/or LCn3PUFA intake/biomarker were presented or such information could be recalculated.

**Figure 1:**
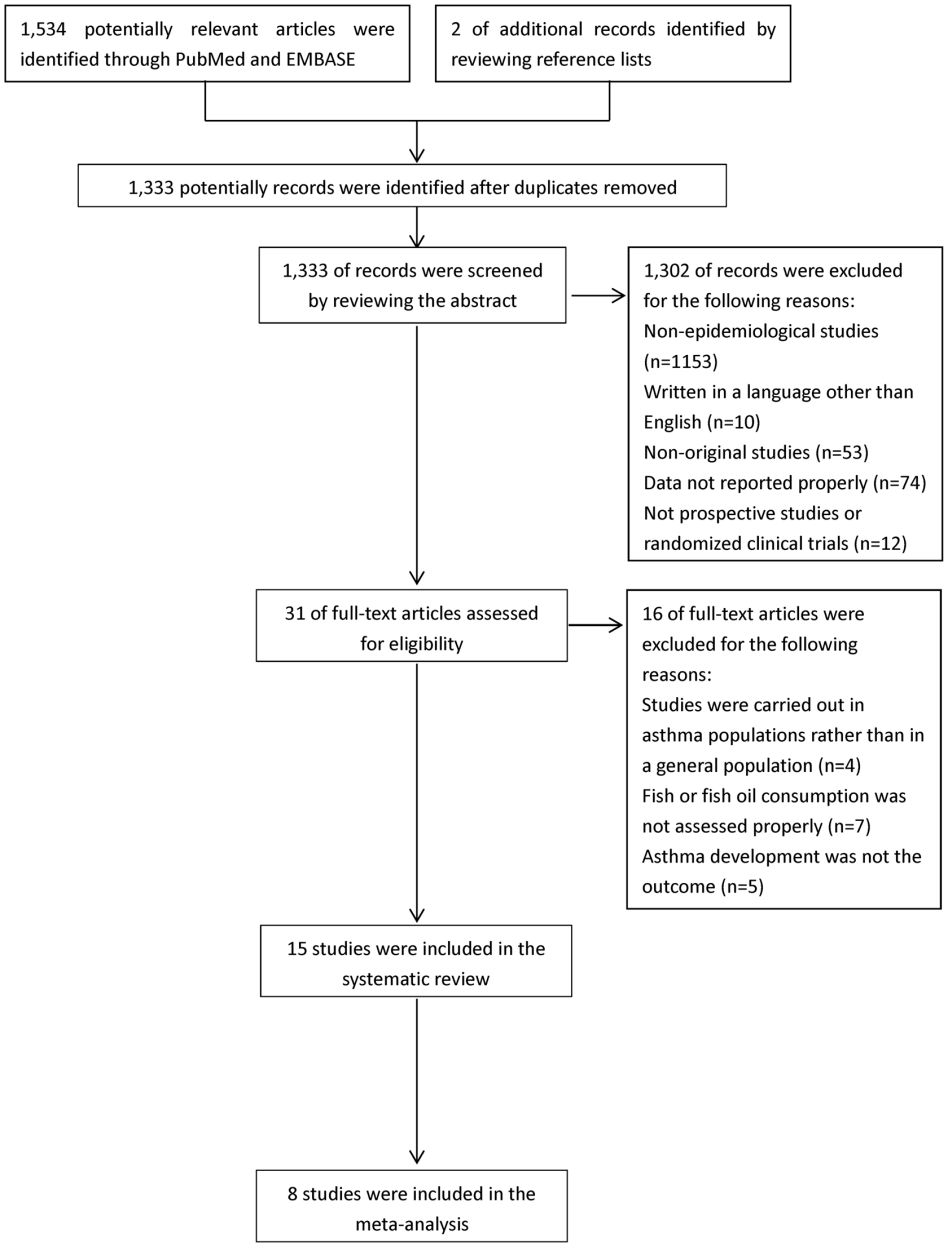
Process of study selection.

As shown in **Figure 1**, of 1,333 non-duplicated abstracts from PubMed and EMBASE, we excluded 1,320 publications because they were non-original studies (e.g., reviews, editorials or letters to editor); were not epidemiological studies; were neither a prospective cohort nor a RCT; were not carried out in a general population; did not report RR for the association between fish or LCn3PUFA intake/biomarker and risk of asthma; did not assess fish or fish consumption properly; did not use the lowest exposure category as the reference; or were not in English. In addition, two articles were identified by reviewing the reference lists. Thus, 15 studies [3], [4], [14]–[17], [21]–[29], including 11 prospective cohorts and 4 RCTs, that reported results on fish or LCn3PUFA intake and risk of asthma were included in this systematic review. Seven studies [4], [21], [22], [24], [26], [27], [29] including 3 prospective cohorts and 4 RCTs _ENREF_3were excluded from the meta-analysis due to the insufficient information for pooling the results.

### Data extraction

The dataset includes the first author’s name, year of publication, study population, country of origin, study design, number of participants, average age at baseline, proportion of males, duration of follow-up, methods for diet measurement, categories of fish or LCn3PUFA intake, case identification methods, number of events, and adjusted covariates, as well as RRs and 95% CIs of asthma risk in the corresponding categories. RRs transformed to their natural logarithms (ln), and the 95% CIs were used to compute the corresponding standard errors (SEs).

### Statistical Analysis

RR was used as a loose term for measuring the association of interest across all primary studies. Hazard ratio and risk ratio were both considered as RR. [30] Because the incidence of asthma in the reference group (*P*_0_) is relatively low, the odds ratio (OR) is also used to approximate RR. We transformed OR to RR by combing the information of *P*_0_ in a sensitivity analysis.

We estimated the pooled RRs and 95% CIs of asthma, comparing the highest to the lowest fish or LCn3PUFA intake using a fixed-effects or random-effects model according to the heterogeneity among studies within each group. In addition, Cochran’s test was used to test for heterogeneity among studies, and *I* ^2^ was computed to determine the degree of inconsistency across studies. Publication bias was assessed by visualizing the funnel plot and determined by the Egger asymmetry test or Begg’s test as appropriate. Sensitivity analysis was conducted to investigate the influence of a single study on the overall estimate.

P≤0.05 was considered statistically significant for all tests. All analyses were performed using STATA statistical software (Version 11.0, STATA Corp, College Station, TX).

## Results

### Study characteristics

The characteristics of the 11 included independent cohorts are shown in **Table 1**. Four of them [4], [15]–[17] examined fish consumption and risk of asthma including 12, 481 children and 996 cases identified during 1 to 6 years of follow-up; three of them were included in the meta-analysis. Two studies [23], [28] reported the association between the LCn3PUFA levels in mothers’ expressed breast milk (EBM) during the breast-feeding period and risk of asthma in 276 infants. Sixty nine cases were diagnosed during a mean follow-up of 5.3 years. The other two studies [26], [29] related risk of asthma in offspring either to maternal fish intake (786 cases/2,832 individuals) or to maternal plasma LCn3PUFA levels (64 cases/951 individuals) during pregnancy with a mean follow-up of 6 and 7 years, respectively. In addition, 3 studies [14], [25], [31] examined fish and/or LCn3PUFA intake and risk of asthma in 82,533 adults including 1,311 incident cases identified during a mean follow-up of 9.9 years.

**Table 1:** Characteristics of included prospective studies.

Author and year of publication	Study name and geographic area	Study design	Participants	Age, year	Men (%)	Duration (average) of follow-up, year	Exposure Assessment	Exposure categories	Asthma assessment	Adjusted variables
**Newborns’ fish exposure and asthma in their childhood**										
*FFQ*										
Nafstad, P. *et al*. 2003	Oslo Birth Cohort Study, Norway	Birth Cohort	2, 531	0	NA	2–4	12-month questionnaire	Early (≤12 m) fish introduction:Yes (1, 080 participants) vs. no (1, 191 participants)	Doctor diagnosed (n = 141)	Parental atopy, atopic eczema 0–6 months of age, gender, parity, birth weight, maternal age at delivery, birth order, uterus-related pregnancy complications, keeping pets at home when the child was born, an episode of lower respiratory tract infections during first year of life, maternal education, family income per year, maternal smoking at the end of pregnancy and length of breastfeeding
Kull, I. *et al*. 2006	BAMSE, Sweden	Birth Cohort	3, 595	0.7	NA	4	FFQ at age 1	Early (≤12 m) fish introduction:never (reference, 348 participants), 1/m (364 participants), 2–3/m (666 participants), 1/w (1275 participants), ≥1/w (942 participants)	At least four episodes of wheezing during the last 12 months or at least one episode of wheezing during the same period, if the child was on inhaled steroids (n = 247)	Parental allergic disease, maternal age, maternal smoking and breastfeeding
Oien, T. *et al*. 2010	Prevention of Allergy among Children in Trondheim, Norway	Birth Cohort	3, 086	Approx. 1	49.5	Approx. 1	FFQ at age 1	Never or <1/w (1493 participants), ≥1/w (1569 participants)	Doctor diagnosed (n = 183)	No adjustment
Willers, S. M. *et al.* 2011	PIAMA, Netherlands	Birth Cohort	3, 269 (subjects with questionnaire information, Ig E data or methacholine provocation test conducted)	2	51.8	6	Annual questionnaires about 30–35 foods or food groups	Fish consumption frequency: continuous, days/week(data of participants for each group is not available)	Parental reported(n = 425)	Gender, maternal education, parental atopy, maternal smoking during pregnancy, smoking in the house at 8 years of age, breast feeding, presence of older siblings, birth weight, overweight mother, overweight child at 8 years of age, geographical region and study arm
*Fatty acids in maternal EBM during breast feeding period and asthma in offspring*										
Wijga, A. H. *et al.* 2006	PIAMA, Netherlands	Birth Cohort	158	0	50.6	4	Breast milk fatty acids concentrations were measured by gas liquid chromatography	EBM fatty acids: continuous, wt% (data of participants for each group is not available)	Parental report (n = 18)	No adjustment
Lowe, A. J. *et al.* 2008	MACS, Australia	Cohort	118	0	NA	7	Breast milk fatty acids concentrations were measured by gas chromatography	EBM n-3 and n-6 fatty acids: continuous, wt% (data of participants for each group is not available)	Parental report or physician diagnosed (n = 51)	No adjustment
**Maternal fish exposure and asthma in their offspring’s childhood**										
Willers, A. M. *et al.* 2008	PIAMA, Netherlands	Birth Cohort	2, 832	1	48.7	7	Questionnaire	Maternal fish consumption frequency during pregnancy: Daily plus regular (973 participants) *vs*. rare consumption (2, 949 participants)	Parental reported (n = 786)	Gender, maternal education, parental allergy, maternal smoking during pregnancy, smoking at home at 8 years of age, breast feeding, presence of older siblings, birth weight, maternal overweight 1 year after pregnancy, maternal supplement use during pregnancy, region and study arm
Notenboom, M. L. *et al*. 2011	KOALA, Netherlands	Birth Cohort	951	0	50.5	6–7	Plasma fatty acids concentrations were determined by gas chromatography	Maternal plasma fatty acids (n-3 long-chain polyunsaturated) concentrations during pregnancy:quintiles with the lowest as reference (data of participants for each group is not available)	Parental report based on doctor diagnosed or medication in the last 12 months at the age of 6–7 years (n = 64)	Recruitment group, maternal age, ethnicity, education level, smoking during pregnancy, parental history of atopy, term of gestation, season of birth, gender, birth weight, mode of delivery, exposure to environmental tobacco, presence of older siblings and sibling atopy, breastfeeding, child day care and pets at home
**Adult’s fish consumption and asthma in their later life**										
Troisi, R. J. *et al.* 1995	NHS, USA	Cohort	77, 866	30–69	0	10	Estimated from semi-quantitative FFQ	n-3 PUFA:quintiles with the lowest as reference (data of participants for each group is not available)	Self-report based on doctor’s diagnosis (n = 760)	Age, smoking, BMI, area of residence, number of physician’s visits and quintiles of energy intake
Nagel G. and Linseisen, J. 2005	EPIC-Heidelberg cohort, Germany	Cohort	525	35–65	35.2	2.1 (median)	Fish consumption was collected by an semi-quantitative FFQ, fish oil was estimated from that	Fish and fish oil consumption:tertiles with the lowest as reference (data of participants for each group is not available)	Doctor diagnosed (n = 105)	Age group, fat energy intake, nonfat energy intake, BMI, smoking status, gender and maternal education
Li J, *et al.* 2012	CARDIA, USA	Cohort	4, 162	24.9	47.0	20	Assessed by interviewer-administered quantitative FFQ	Fish and n-3 PUFA:Quintiles with the lowest as reference (877, 800, 822, 829 and 834 participants for quintile groups, divided by n-3 PUFAs consumption, from lowest to highest)	Self-reported physician diagnosis and/or asthma-control medicine (n = 446)	Age (continuous), gender, race, study center, education, smoking status, alcohol consumption, physical activity, BMI, total energy intake, dietary intake of linoleic acid

Abbreviations: FFQ, Food Frequency Questionnaire; NA, Not Available; BAMSE, Barn/Children, Allergy/Asthma, Milieu, Stockholm, Epidemiologic; PIAMA, Prevention and Incidence of Asthma and Mite Allergy; EBM, Expressed Breast Milk; MACS, Melbourne Atopy Cohort Study; KOALA, Child, Parent and Health: Lifestyle and Genetic Constitution; NHS, Nurses’ Health Study; BMI, Body Mass Index; EPIC, European Prospective Investigation into Cancer and Nutrition; CARDIA, Coronary Artery Risk Development in Young Adults.

We also included randomized controlled trials (RCTs) [21], [22], [24], [27] in this systematic review. A total of 1, 063 participants with 607 participants in the fish oil treatment group were involved in (**Table 2**).

**Table 2:** Characteristics of included randomized controlled trials (RCTs).

Author and year of publication	Study name and geographic area	Participants	Age, year	Men (%)	Duration (average) of follow-up, year	Exposure Assessment	Exposure categories	Asthma assessment
Dunstan J. A., *et al.* 2003	Subiaca, Western Australia	83	0	NA	1	Fish oil supplementation containing 3.7 g/d of n-3 PUFAs from 20 weeks of pregnancy till delivery	Maternal fish oil supplement (40 participants) *vs*. olive oil placebo (43 participants)	Infants were clinically evaluated for asthma (8 cases)
Lauritzen L., *et al.* 2005	Dnaish National Birth Cohort, Danish	65	0	59.1	2.5	Fish oil supplementation containing 1.5 g/d of n-3 PUFAs for the first 4 months of lactation	Maternal fish oil supplement (37 participants) *vs.* olive oil placebo (28 participants)	Parental report
Marks G. B., *et al.* 2006	Sydney, Australia	516	0	NA	5	High *vs.* low n-3 PUFAs supplementation since bottle feeding or solid foods, whichever came first	High n-3 PUFAs supplementation (267 participants) and low n-3 PUFAs supplementation (249 participants)	Parental report
Olsen S. F., *et al.* 2008	Aarhusm Denmark	399	0	NA	16	Fish oil supplementation containing 2.7 g/d of n-3 PUFAs were given to pregnant women since 30 weeks of gestation till delivery	Maternal fish oil supplement (263 participants) *vs*. olive oil placebo (136 participants)	Doctor diagnosed

Abbreviations: NA, Not Available; PUFA, Polyunsaturated fatty acids.

### Infants’ fish consumption and risk of asthma in childhood

**Figure 2:**
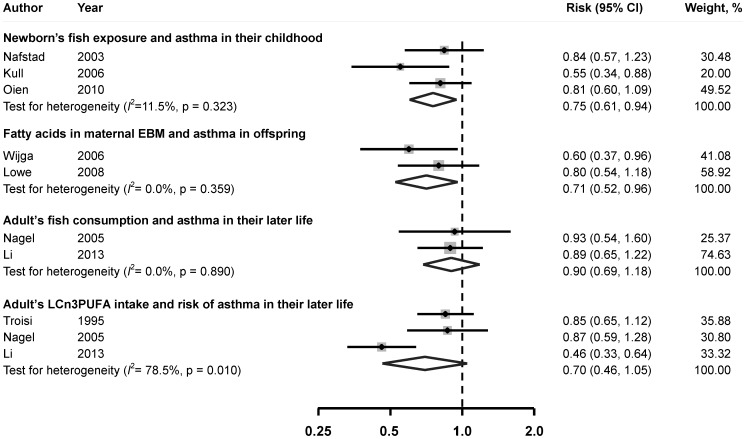
Multivariable adjusted relative risk and 95% confidence interval of risk of asthma. The pooled estimates were obtained using a fixed-effects or random-effects model depending on the heterogeity test. The dots indicate the adjusted RRs by comparing highest vs. lowest of the exposure of interest. The size of the shade square is proportional to the percent weight of each study. The horizontal lines represent 95% CIs. The diamond data markers indicate the pooled RRs. Abbreaviations:CI, confidence interval; EBM, Expressed Breast Milk; LCn3PUFA: long-chain n-3 polyunsaturated fatty acid; RR, relative risk.

Meta-analysis suggested that fish consumption in infants was inversely associated with the incidence of asthma in their childhood. The pooled RR of asthma was 0.76 (95% CI, 0.61–0.94), comparing the highest to the lowest category of fish consumption (**Figure 2**). No substantial heterogeneity was observed across studies (*I* ^2^ = 11.5%, *P* = 0.32).

### LCn3PUFA in maternal EBM during breast feeding period and risk of asthma in offspring

LCn3PUFA levels in maternal EBM were found to be inversely associated with the incidence of asthma in offspring. The combined RR was 0.71 (95% CI, 0.52–0.96), comparing the highest to the lowest group of LCn3PUFA levels (**Figure 2**). No significant heterogeneity was observed across studies (*I* ^2^ = 0.0%, *P* = 0.36).

### Maternal fish consumption and risk of asthma in offspring

Because the exposures in these two studies were not the same, the results cannot be pooled. In Willers’s study [29], the maternal fish consumption frequency during pregnancy was not related to asthma development in offspring, the OR of asthma was 1.01 (95% CI, 0.85–1.20) comparing individuals who consumed fish ≥ 1/week with those ate fish < 1/week. Consistently, Notenboom *et al*. [26] reported that maternal plasma LCn3PUFA concentration was not related to the risk of asthma in offspring.

### Fish consumption and risk of asthma in adults

Comparing those in the highest group of fish consumption with those in the lowest group, no significant association was found between fish and asthma. The pooled RR of incidence of asthma was 0.90 (95% CI: 0.69–1.18). Significant heterogeneity among studies was not found (*I* ^2^ = 0.0%, *P* = 0.89). Similar results were observed for LCn3PUFA intake (RR: 0.70, 95% CI, 0.46–1.05; *I^2^* = 78.5%, *P* = 0.01).

### Sensitivity analysis

Omitting 1 study each time and recalculating the pooled RRs for the remainder of the studies show that none of the individual studies substantially influenced the results. Considering the information of incidence of asthma in the reference group when transforming the ORs into RRs, the results were not materially changed.

### Fish oil supplementation and asthma in children

Only one study [27] found maternal LCn3PUFA supplementation to be beneficial for offspring’s asthma risk, however, results from other trials were inconsistent. To date, findings from clinic trials do not support that LCn3PUFA supplementation is beneficial in terms of asthma prevention in childhood (**Table 2**).

## Discussion

By pooling data from published prospective cohort studies on the association of fish consumption or LCn3PUFA intake/biomarker and risk of asthma, we found that intake of fish or LCn3PUFAs was significantly inversely related to the risk of asthma in children based on the available literature. This inverse association was attenuated in adults.

Laboratory studies suggested that LCn3PUFAs might have the ability to inhibit the production of prostaglandin E_2_ (derived from arachidonic acid), suppress T-helper 2 (Th2) cell’s response to allergens [32]_ENREF_6, and consequently modulate the intensity and duration of inflammatory responses [33], [34]. Thus, it was hypothesized that the increased intake of LCn3PUFAs can reduce the risk of atopic diseases such as asthma [32]. In the present meta-analysis, we found that the potential beneficial effect of fish or LCn3PUFA intake was more pronounced in children. The mechanism for the difference between children and adults is unclear. It might be explained by the suggestion that children are more sensitive to LCn3PUFAs. Studies suggest that chronic inflammation may affect the immune system [1]. It may be particularly true in children because a child’s immune system is under development [35]. Presumably, LCn3PUFAs are important in the stage of immune system development. In other words, children may be more sensitive to LCn3PUFA intake than adults. Nevertheless, further studies are needed.

Findings from other types of epidemiological studies on fish or LCn3PUFA and asthma are not so consistent. Two cross-sectional studies [36], [37] found an inverse association: one observed a 46% risk reduction in doctor-diagnosed asthma in children with per unit increment of fish consumption [36]; and the other one reported that the risk of asthma reduced by 68% comparing those in the highest with those in the lowest tertile of fish consumption [37]. However, another cross-sectional study found a positive association of fish consumption with the risk of asthma [19]. In addition, one case-control study found a non-significant risk reduction comparing the highest fish consumption group with the lowest [18]. Moreover, a meta-analysis summarized 9 _ENREF_33randomized controlled trials (RCTs) and found that LCn3PUFA supplementation was not associated with improved asthma symptoms [38], both in children and adults. Another meta-analysis on RCTs [39] also reported a non-significant inverse association between fish oil supplementation and asthma risk in children. The null findings were in concordance with our results in adults. Of note, all included RCTs in that meta-analysis [38] had relatively small sample sizes (from 12 to 45) and short follow-up periods (from 8 weeks to 12 months). Also, in RCTs, fish oil supplementation was used, which may reflect a different health impact from consuming whole fish – a package of nutrients [40]. Thus, our meta-analysis of cohort studies, coupled with the other meta-analysis of RCTs, provided important evidence for future research and primary prevention of asthma.

Since our meta-analysis is based on observational studies, the inherent limitations of primary studies may have affected our findings. For example, the possibility of residual confounding cannot be ruled out. In addition, although we identified 11 cohort studies on this topic, these studies had to be divided into 3 subgroups because of the various exposure measurements. Nevertheless, our results should not be substantially biased given the potential biological mechanisms and the consistence with findings from RCTs.

In conclusion, our pooled analysis suggests that intake of fish or LCn3PUFAs in expectant mothers or infants is inversely associated with asthma development, particularly, in childhood. This meta-analysis adds evidence to the literature that fish, as a general healthy food, should be recommended to pregnant women and infants, though certain types of fish with high level of contaminants need to be avoided.

## Supporting Information

Checklist S1 PRISMA 2009 Checklist.
(DOC)
